# Evaluation of primary physicians’ awareness regarding the radiological findings of osteosarcoma: A retrospective study

**DOI:** 10.1097/MD.0000000000042682

**Published:** 2025-06-06

**Authors:** Manabu Hoshi, Naoto Oebisu, Tadashu Iwai, Naoki Takada, Yoshitaka Ban, Masanari Aono, Hiroaki Nakamura

**Affiliations:** a Department of Orthopedic Surgery, Osaka City General Hospital, Osaka, Japan; b Department of Orthopedic Surgery, Osaka Metropolitan University Graduate School of Medicine, Osaka, Japan.

**Keywords:** osteosarcoma, physician awareness, radiology, referral document

## Abstract

A referral from a primary physician is a crucial initial diagnostic intervention for the management of osteosarcoma. We reviewed the awareness of primary physicians regarding the radiological findings of osteosarcoma through their referral documents. Referral documents of 40 patients (27 male and 13 female; median age, 19 [8–81] years) with osteosarcoma were retrospectively investigated. The documents were examined for suggested supplementary radiological procedures, presence or absence of a descriptive keyword for the radiological findings of osteosarcoma, periosteal reaction, matrix, soft tissue mass, type of border of the lesion, and type of bone destruction, followed by an assessment of the diagnostic radiologists’ findings. The keywords “periosteal reaction,” “matrix,” “soft tissue extension,” “border of the lesion,” and “type of bone destruction” were described in 5 (7.5%), 5 (15%), 4 (10%), 0 (0%), and 7 (17.5%) cases by the primary physicians, respectively. Records with “periosteal reaction,” “matrix,” “soft tissue mass,” “type of border of the lesion,” and “type of bone destruction” were described in 5 (31.25%), 5 (31.25%), 10 (62.5%), 3 (18.8%), and 3 (18.8%) radiologic findings of the diagnostic radiologists, respectively. The descriptions of keywords for radiological findings suggesting osteosarcoma in referral documents from primary physicians seemed generally insufficient. More enhanced and comprehensive awareness of the typical radiological findings of osteosarcoma is necessary to prevent diagnostic delays.

## 1. Introduction

**Figure 1.**
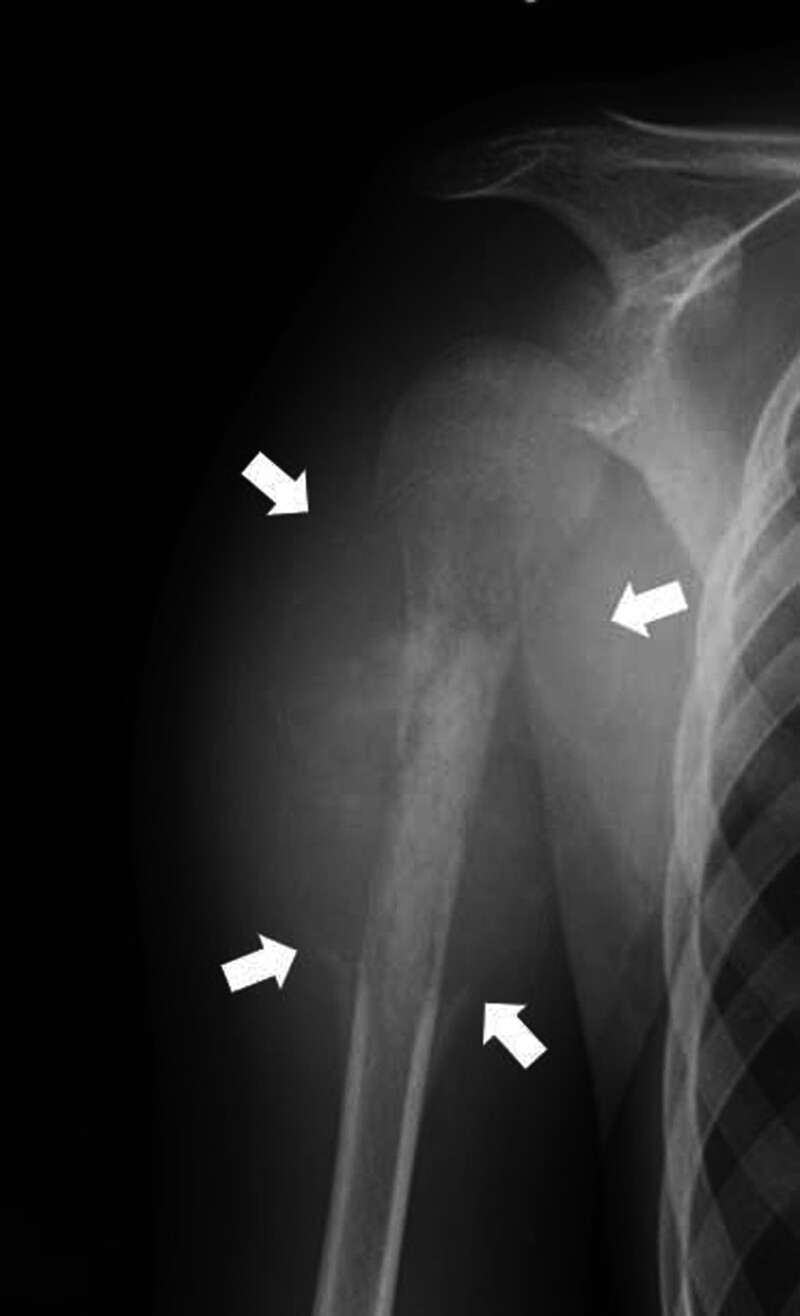
Plain radiograph of a 12-yr-old girl with osteoblastic osteosarcoma in the right proximal humerus. The periosteal reaction of sunburst appearance/spicula/Codman’s triangle, matrix of abnormal osteoblastic change and new bone formation, soft tissue extension, ill-defined border, and permeative-type bone destruction were observed. CT = computed tomography, MRI = magnetic resonance imaging.

Despite recent advancements in various radiological modalities, explicit initial radiological information remains an important step in the diagnosis of primary malignant bone tumors because it provides vital information about the site, multiplicity, periosteal reaction, matrix, soft tissue extension, type of lesion border, and bone destruction.^[1,2]^ Osteosarcoma is a common primary malignant bone tumor that arises in adolescent and young adult patients; however, its incidence is very rare.^[3–5]^ With regard to the description of periosteal reactions, the keywords “sunburst appearance,” “spicula,” and “Codman’s triangle” are representative radiological findings of primary malignant bone tumors of osteosarcoma (Fig. 1).

Primary physicians should be familiar with these keywords to describe the radiological findings of osteosarcoma. When awareness of the radiological findings of osteosarcoma by primary physicians is low, diagnostic delay may have a negative impact on final survival.^[6]^ However, in routine medical practice, it is unclear whether primary physicians can appropriately use these important keywords to identify osteosarcomas when encountering this tumor. To the best of our knowledge, no prior studies have elucidated primary physicians’ awareness of the use of accurate keywords for describing osteosarcomas. In this study, we reviewed whether these important keywords, suggestive of osteosarcoma, were described in patient referral documents by attending physicians.

## 2. Materials and methods

### 2.1. Study design and patient selection

The inclusion criteria were as follows: patients with a pathological diagnosis of osteosarcoma; patients whose medical records at the initial visit were available; and (3) patients whose referral documents from primary physicians could be assessed.

Forty patients diagnosed with osteosarcoma between September 2005 and December 2022 were selected from an institutional database. We collected data from medical records, including demographic details such as age, sex, stage, affiliated institution (clinic or hospital), specialty of previous physicians, histological subtype of osteosarcoma, and affected site. All diagnoses were confirmed according to the World Health Organization criteria.^[7]^ The clinical stage of each patient was evaluated according to the guidelines of the American Joint Committee on Cancer for bone tumors.^[8]^ In this study, a clinic in Japan was defined as a facility where patients receive medical advice or treatment, mostly composed of a small number of staff, with <20 beds. A hospital is a medical institution with multiple departments and 20 beds.

### 2.2. Evaluation of primary physician’s awareness

In patient referral documents, to evaluate the primary physician’s awareness of osteosarcoma, the presence and/or absence of descriptive keywords for radiological investigations suggesting osteosarcoma were assessed,^[1,9]^ in addition to the description of clinical symptoms. These included periosteal reaction (sunburst appearance, spicula, Codman’s triangle, or other abnormal findings), matrix (composition of the tumor tissue, abnormal osteoblastic change, and new bone formation), soft tissue extension/mass, type of border of the lesion (sharp sclerotic, sharp lytic, or ill-defined), and type of bone destruction (geographic, moth-eaten, or permeative pattern; Fig. 1).

Additionally, the presence or absence of associated radiological information on plain radiography, magnetic resonance imaging (MRI), computed tomography (CT), laboratory data, and the interpretation of such findings by diagnostic radiologists were examined. We assessed and compared the description rates of keywords used by diagnostic radiologists for radiological findings with those used by primary physicians.

Pathological fractures sometimes indicate the clinical onset of osteosarcoma. The presence or absence of descriptive radiological findings at the first visit to our institution were also investigated in the patients’ referral documents.

### 2.3. Ethical considerations

This study was performed in accordance with relevant guidelines and regulations and approved by the Institutional Review Board of the Osaka Metropolitan University Graduate School of Medicine. This study was a retrospective chart review; thus, consent for participation was waived, and approval of this waiver was obtained by the Institutional Review Board of Osaka Metropolitan University Hospital.

### 2.4. Statistical analyses

Fisher’s exact probability test was performed to statistically compare the description rates of the keywords by primary physicians’ and diagnostic radiologists. ’ Statistical significance was set at *P* < .05, and analyses were performed using Excel Statistics for Windows (version 2025; SSRI Co., Ltd., Tokyo, Japan).

## 3. Results

We included 40 patients (27 male and 13 female), and their clinical characteristics are listed in Table 1. The median age at diagnosis was 19 years (range: 8–81 years). The (tumor, node, metastasis) TNM staging was as follows: stage IIA, 3 cases; stage IIB, 26 cases; stage III, 1 case; stage IVA, 9 cases; and stage IVB, 1 case.

**Table 1:** Demographic data of the patients with osteosarcomas.

Factors		Number	
Age	Years (Mean)	19 yr.	8–81 yr
Sex	Male	27	67.5%
	Female	13	32.5%
TNM staging	IIA	3	7.5%
	IIB	26	65.0%
	III	1	2.5%
	IVA	9	22.5%
	IVB	1	2.5%
Affiliated institution of previous physicians	Hospital	30	75.0%
	Clinic	10	25.0%
Specialty of previous physicians	Orthopedic surgeon	37	92.5%
	Orthopedic oncologist	4	10.0%
	General surgeon	1	2.5%
	Medical oncologist	1	2.5%
	Plastic surgeon	1	2.5%
Subtype	Osteoblastic	18	45.0%
	Fibroblastic	12	30.0%
	Chondroblastic	6	15.0%
	Small cell	2	5.0%
	Telangiectatic	1	2.5%
	High grade surface	1	2.5%
Affected site	Femur	21	52.5%
	Humerus	8	20.0%
	Tibia	6	15.0%
	Pelvis	2	5.0%
	Scapula	1	2.5%
	Spine	1	2.5%
	Sternum	1	2.5%
Pathological fracture	Yes	5	12.5%
	None	35	87.5%

The types of institutions affiliated with the primary physicians were hospitals in 30 (75.0%) and clinics in 10 (25.0%) cases. The primary physicians were mostly composed of orthopedic surgeons in 37 cases (92.5%), including 4 certified orthopedic oncologists. In Japan, to work as a certified orthopedic surgeon, an experience of 4 years or more and completion of a board examination are required. Moreover, to practice as a certified orthopedic oncologist, several requirements must be fulfilled, including the completion of certification for specialists in the medical field, palliative care training sessions, more than 2 years of full-time experience in a cancer treatment institution designated by the Japanese Board of Cancer Therapy, experience in treating more than 20 cancer cases, and conference and paper presentations.

Osteosarcoma subtypes are mainly of the conventional type. The main affected sites were the femur, humerus, and tibia. The incidence of pathological fractures was 12.5% (5 of 40). Clinical symptoms, as described in the patients’ referral documents by primary physicians, were also investigated. Pain was common in 24 cases, palpable mass in 3, limb dysfunction in 1, discomfort in the affected limb in 1, and no description in 14 cases.

**Figure 2.**
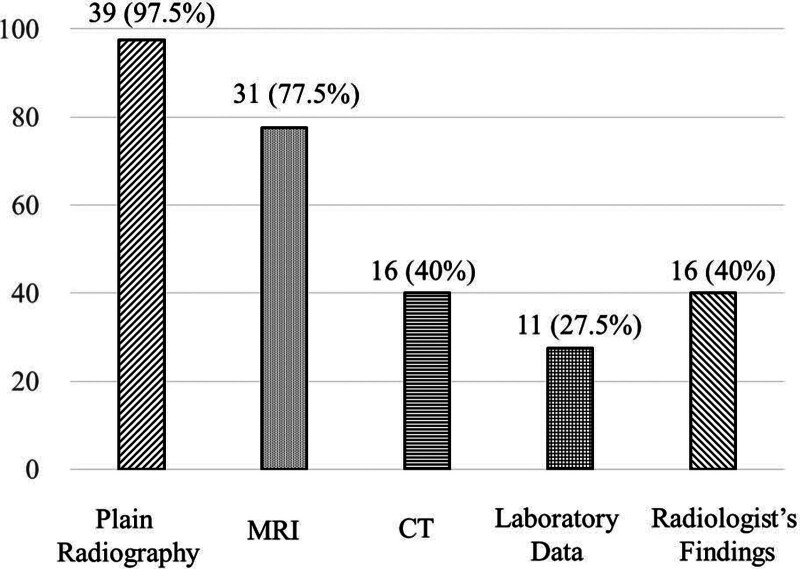
Imaging modalities in addition to referral documents.

The 40 patient referral documents revealed that most patients underwent plain radiography and MRI and a small number of the patients (28%) underwent laboratory tests (Fig. 2). Furthermore, 16 (40%) referral documents were augmented to the radiologists’ interpretations. Radiological modalities included plain radiography only in 6 patients, plain radiography and CT in 3, plain radiography and MRI in 17, plain radiography, CT, and MRI in 13, and MRI only in 1. Laboratory tests were conducted in 11 (28%) of 40 patients.

To monitor osteosarcoma awareness, we investigated 5 keywords that suggested osteosarcoma in 40 referral documents (Table 2). The keywords “sunburst appearance,” “spicula,” and “Codman’s triangle” Codman’s triangle were extremely rare. A small number of primary physicians commented on the matrix, indicating “abnormal osteoblastic changes, new bone formation, soft tissue extension/mass, and the type of bone descent.

**Table 2:** Comments of radiological findings of osteosarcoma.

	Primary physicians (n = 40)	(%)	Radiologists (n = 16)	(%)	*P*-value
Periosteal reaction					
Sunburst appearance	0	0	0	0	.04
Spicula	1	2.5	2	13	
Codman’s triangle	0	0	1	6.3	
Others	2	5	2	13	
Matrix	6	15	5	31	.26
Soft tissue extension/mass	4	10	10	63	.001
Border	0	0	3	19	.02
Bone destruction	7	18	3	19	.59

We assessed diagnostic radiologists’ findings in 16 patients by examining the same 5 keywords. Only a few radiologists commented on the abnormal matrix, periosteal reactions, type of border, and type of bone destruction. Interestingly, 10 (62.5%) radiologists commented on soft tissue extensions/masses. With regard to keywords suggesting malignant bone tumors, diagnostic radiologists described periosteal reaction (*P = *.04), soft tissue mass/extension (*P* = .001), and type of lesion border (*P* = .02) more frequently than did primary physicians. Three primary physicians (60%) and 4 radiologists (80%) commented on the pathological fractures underlying the 5 osteosarcoma subtypes.

## 4. Discussion

Osteosarcoma are a common primary malignant bone tumor that frequently occurs in adolescents and young adults. Healthcare workers and even the general population are acquainted with the term “osteosarcoma”; moreover, knowledge regarding keywords like “sunburst appearance,” “spicula,” and “Codman’s triangle” describing osteosarcoma have been imparted in medical school textbooks. These terms also constitute frequently asked questions for doctors undertaking national board examinations. Furthermore, to qualify as practicing orthopedic surgeons and diagnostic radiologists, doctors must deepen their knowledge of typical radiological interpretations. However, in this study, no physician or radiologist commented on the “sunburst appearance,” and only 1 radiologist commented on “Codman’s triangle” in the referral documents of patients with osteosarcoma.

This study revealed that descriptive keywords for osteosarcoma were lacking in primary physicians’ referral documents, which sometimes led to diagnostic delays. This can be attributed to the rarity of osteosarcoma and its incidence (<1% of all cancers).^[3–5]^ A previous study reported that general physicians rarely encounter patients with primary malignant bone tumors, estimating <0.7 cases of carriers are over 10 years of age,^[10]^ indicating a lack of experience in diagnosing osteosarcoma. Second, it was reported that primary physicians have limited familiarity with sarcoma guidelines and have the propensity to advise patients to “watch and wait” for severely enhanced symptoms.^[10,11]^

Clinical symptoms are the 1st important step in the diagnosis of bone tumors. In this study, pain was common in 24 patients. Physicians tend to falsely believe that clinical features are temporary. However, when patients complain of pain at rest, worsening pain even after taking painkillers, and pain progressing over time without any minor injury to the affected limbs, physicians should start a comprehensive examination, considering the possibility of bone tumors.^[12,13]^ Additionally, in a previous study, a palpable mass in a patient with osteosarcoma was considered a vital symptom and was observed in more than one-third of patients at the 1st visit.^[14]^ These results emphasize the importance of careful consideration of the patient history and comprehensive physical examination of the affected limb.

Typical osteosarcomas demonstrate radiological features of permeative or moth-eaten bone destruction with ill-defined borders, presenting with tumor-bone cloud-like opacities, aggressive periosteal reactions, and soft tissue extension. However, in clinical practice, it is sometimes difficult to suspect such rare bone tumors and the extent to which general physicians pay attention to these findings remains unclear. We reevaluated the referral documents of the patients and investigated 5 typical findings.

The output of information on periosteal reaction, matrix, soft tissue mass/extension, type of border of the lesion, and type of bone destruction can aid referring physicians in accurately conveying the possibility of malignancy to orthopedic oncologists, thereby preventing diagnostic delays.^[15,16]^ However, only 5 of 40 (12.5%) primary physicians commented on periosteal reactions, 6 (15%) on matrix, 4 (10%) on soft tissue mass/extension, and 7 (17.5%) on bone destruction. These results revealed that dissemination of important radiological information concerning osteosarcoma from the primary physician was insufficient.

Plain radiography provides significant basic information about bone tumors, and CT/MRI is an indispensable technique for evaluating tumor features.^[17]^ This study also showed that patients underwent local MRI (77.5%) and CT (40%) in addition to plain radiography (97.5%), proving MRI is the preferred imaging modality. Notably, in this study, 16 of the 40 previous physicians (40%) spontaneously added “radiologists’ findings” as additional information. This result suggests that previous physicians lacked confidence in the radiological diagnosis of osteosarcoma, or they hoped to enhance the accuracy of their diagnosis after cross-examining their findings.

Notably, 37 of the previous 40 physicians (92.5%) were orthopedic surgeons, indicating that most patients with osteosarcoma visited orthopedic surgeons. Therefore, knowledge of the radiological findings of osteosarcoma is prevalent among orthopedic surgeons. Diagnostic radiologists reported these comments more frequently, particularly in terms of the periosteal reaction (*P* = .04), soft tissue mass/extension (*P* = .001), and border type (*P* = .02). This result suggests that the diagnostic radiologists were well-educated and aware of osteosarcoma diagnosis. Training is necessary during the initial stages for young medical doctors. Moreover, awareness of the information output of disease-specific findings must always be enhanced in medical conferences, especially orthopedic-associated conferences, and continuous medical education is absolutely necessary.^[18]^

Laboratory data is one of the most helpful modalities for diagnosing osteosarcoma. About 46% of osteosarcoma cases have elevated alkaline phosphatase values,^[19]^ and early testing in primary hospitals/clinics also seems to reflect timely osteosarcoma diagnosis. In this study, 11 (28%) cases underwent laboratory tests. Kotrych et al^[11]^ reported that laboratory tests was carried out only in 15% of cases diagnosed the osteosarcoma.

Pathological fractures are sometimes indicative of the clinical onset of osteosarcoma. Generally, pathological fractures occur in 5% to 10% of patients with osteosarcoma.^[20,21]^ In this study, 5 patients (12.5%) presented with pathological fractures in their affected limbs. Three primary physicians (60%) and 4 radiologists (80%) identified pathological fractures, indicating a high notification rate of osteosarcoma. Compared to other radiological findings, such as periosteal reaction and border type, primary physicians and radiologists were more familiar with pathological fractures.

The limitation of this study is its retrospective nature, and it was performed in a specific region of Japan. The proportion of patients with osteosarcoma was relatively small. Second, only the actual output of information in the referral documents was reviewed; thus, the presence or absence of direct consultations between previous physicians and orthopedic oncologists and the background and experience of primary physicians could not be assessed. Third, this study included not only patients with conventional osteosarcoma but also those with small-cell, telangiectatic, and high-grade surface osteosarcoma, and these subtypes may be considered to be different from the typical radiological findings of conventional osteosarcoma. Fourth, none of the cases exhibited typical imaging findings of osteosarcoma, and each case was variable.^[22]^

In conclusion, the description rates of these keywords were insufficient. However, diagnostic radiologists added more key findings than previous physicians, mainly orthopedic surgeons. Therefore, awareness regarding the radiological findings of osteosarcoma should be enhanced, and better communication for relaying important keywords in referral documents is required for high diagnostic accuracy, thereby preventing diagnostic delays in osteosarcoma.

## Author contributions

**Conceptualization:** Manabu Hoshi.

**Data curation:** Naoto Oebisu.

**Formal analysis:** Naoki Takada, Yoshitaka Ban.

**Software:** Tadashu Iwai.

**Supervision:** Hiroaki Nakamura.

**Writing – review & editing:** Masanari Aono.

**Writing – original draft:** Manabu Hoshi.

## References

[1] Greenspan A Remagen W. Diffential diagnosis of tumors and tumor-like lesions of bones and joints, Radiologic and pathologic approach to bone tumors, 1st ed. Philadelphia: Lippincott-raven Publishers; 1998:1–24.

[2] Uni KK. Dahlin’s Bone tumors. General aspects and data on 11087 cases. 5th ed. Philadelphia, NY: Lippincott-raven Publishers. Osteosarcoma; 1996: 143–83.

[3] Mirabello L Troisi RJ Savage SA. Osteosarcoma incidence and survival rates from 1973 to 2004: data from the Surveillance, epidemiology, and end results program. Cancer. 2009;115:1531–43. 19197972 10.1002/cncr.24121 PMC2813207

[4] Ottaviani G Jaffe N. The epidemiology of osteosarcoma. Cancer Treat Res. 2009;152:3–13. 20213383 10.1007/978-1-4419-0284-9_1

[5] Siegel RL Miller KD Wagle NS Jemal A. Cancer statistics, 2023. CA Cancer J Clin. 2023;73:17–48. 36633525 10.3322/caac.21763

[6] Soomers V Husson O Young R Desar I Van der Graaf W. The sarcoma diagnostic interval: a systematic review on length, contributing factors and patient outcomes. ESMO Open. 2020;5:e000592. 32079621 10.1136/esmoopen-2019-000592 PMC7046415

[7] Baumhoer D Hogendoom PCW Bohlling TO. WHO Classification of Tumors of Soft Tissue and Bone. 5th ed. International Agency for Research on Cancer, Lyon, 2020. Osteosarcoma 2020;403–409.

[8] Frederick LG David LP Irvin DF. AJCC Cancer Staging Manual. 8th ed. Springer. Bone. 2017:187–92.

[9] Matcuk GR Jr Waldman LE Fields BKK. Conventional radiography for the assessment of focal bone lesions of the appendicular skeleton: fundamental concepts in the modern imaging era. Skeletal Radiol. 2024;54:1391–406. 39718620 10.1007/s00256-024-04854-6 PMC12078366

[10] Fossum CC Breen WG Sun PY Retzlaff AA Okuno SH. Assessment of familiarity with work-up guidelines for bone and soft tissue sarcoma among primary care practitioners in Minnesota. Mayo Clin Proc Innov Qual Outcomes. 2020;4:143–9. 32280924 10.1016/j.mayocpiqo.2019.12.002 PMC7140016

[11] Kotrych D Ciechanowicz D Pawlik J. Delay in diagnosis and treatment of primary bone tumors. Ortop Traumatol Rehabil. 2023;25:9–22. 38078348 10.5604/01.3001.0053.4026

[12] George A Grimer R. Early symptoms of bone and soft tissue sarcomas: could they be diagnosed earlier. Ann R Coll Surg Engl. 2012;94:261–6. 22613305 10.1308/003588412X13171221590016 PMC3957506

[13] Dyrop HB Safwat A Vedsted P. Characteristics of 64 sarcoma patients referred to a sarcoma center after unplanned excision. J Surg Oncol. 2016;113:235–9. 26776152 10.1002/jso.24137

[14] Widhe B Widhe T. Initial symptoms and clinical features in osteosarcoma and Ewing sarcoma. J Bone Joint Surg Am. 2000;82:667–74. 10819277 10.2106/00004623-200005000-00007

[15] Vasquez L Silva J Chavez S. Prognostic impact of diagnostic and treatment delays in children with osteosarcoma. Pediatr Blood Cancer. 2020;67:e28180. 31925940 10.1002/pbc.28180

[16] Vasquez L Silva J Chavez S Zapata A. Delay in diagnosis of primary osteosarcoma of bone in children: have we improved in the last 15 years and what is the impact of delay on diagnosis? J Bone Oncol. 2021;29:100359. 10.1016/j.jbo.2021.100359 PMC8056435 33898215

[17] Strauss SJ Frezza AM Abecassis N. Bone sarcomas: ESMO-EURACAN-GENTURIS-ERN PaedCan Clinical Practice Guideline for diagnosis, treatment and follow-up. Ann Oncol. 2021;32:1520–36. 34500044 10.1016/j.annonc.2021.08.1995

[18] Guerra RB Tostes MD da Costa Miranda L. Comparative analysis between osteosarcoma and Ewing’s sarcoma: evaluation of the time from onset of signs and symptoms until diagnosis. Clinics (Sao Paulo). 2006;61:99–106. 16680325 10.1590/s1807-59322006000200003

[19] Bacci G Longhi A Ferrari S. Prognostic significance of serum alkaline phosphatase in osteosarcoma of the extremity treated with neoadjuvant chemotherapy: recent experience at Rizzoli Institute. Oncol Rep. 2002;9:171–5. 11748477

[20] Gonzalez MR Bedi A Karczewski D Lozano-Calderon SA. Are Pathologic Fractures in patients with osteosarcoma associated with worse survival outcomes? A systematic review and meta-analysis. Clin Orthop Relat Res. 2023;481:2433–43. 37184541 10.1097/CORR.0000000000002687 PMC10642876

[21] Kelley LM Schlegel M Hecker-Nolting S. Pathological fracture and prognosis of high-grade osteosarcoma of the extremities: an analysis of 2,847 Consecutive Cooperative Osteosarcoma Study Group (COSS) Patients. J Clin Oncol. 2020;38:823–33. 31928458 10.1200/JCO.19.00827

[22] Crombé A Simonetti M Longhi A Hauger O Fadli D Spinnato P. Imaging of osteosarcoma: presenting findings, metastatic patterns, and features related to prognosis. J Clin Med. 2024;13:5710. 39407770 10.3390/jcm13195710 PMC11477067

